# Professional Role Identity: At the Heart of Medical Collaboration Across Organisational Boundaries

**DOI:** 10.5334/ijic.4184

**Published:** 2019-04-02

**Authors:** Nassera Touati, Charo Rodríguez, Marie-Andrée Paquette, Lara Maillet, Jean-Louis Denis

**Affiliations:** 1 École Nationale d’Administration Publique (ENAP), Québec, CA; 2 Department of Family Medicine, Faculty of Medicine, McGill University, Quebec, CA; 3 Action Center for Prevention and Rehabilitation of Disabilities at Work, University of Sherbrooke, CA; 4 School of public health, University of Montreal, CA

**Keywords:** professional identity, collaboration, physicians, case study

## Abstract

**Purpose:**

The purpose of this paper was to help answer two persistent calls in the literature: the first asks to strengthen the understanding of medical collaboration across levels of healthcare delivery; the second one requests paying more attention to the individual experience of different forms of professional work. Accordingly, the study was guided by the following research question: How do family physicians and specialists working at different levels of healthcare delivery enact their professional identity when interacting in their situated clinical contexts?

**Methodology:**

This was a multiple interpretive case study in which, based on Giddens’ ideas, professional identity was viewed as a dynamic structural element of social life recursively related to professionals’ collaborative actions through sensemaking processes. The study involved 57 participants. Face-to-face individual semi-structured interviews and organizational documents were the main sources of data. Deductive-inductive thematic analysis was adopted as strategy for data analysis.

**Findings:**

Three prevailing physicians’ identity roles were elicited: medical expert, care coordinator, and team member. These professional identities, not mutually exclusive, were instantiated in three specific modalities of collaboration: quasi-inexistent, restrained, and extended. The entanglement of a particular identity role and a specific collaborative practice became meaningful through a complex net of organizational and institutional features, and patients’ nosological profiles.

## Introduction

Change towards collaboration has been a pervasive trend in many institutional fields as well as in the healthcare sector. Most current health reforms in Western countries have in fact explicitly encouraged both inter-organizational and interprofessional collaboration as privileged means to achieve effectiveness and efficiency in the delivery of care and, ultimately, warrant the sustainability of healthcare systems [1,2].

At the same time, professions, which are institutions themselves, have also experienced significant changes over the last decades [3,4]. As noted by Adler and Kown: “A growing literature suggests that the Anglo-American institution of professionalism – understood both as type of occupation (‘the profession’) and as type of individual work identity (‘the professional’) – is in the process of a profound and contested mutation” [3, p. 930]. Framed by ubiquitous market logics and discourses of efficiency, professionals are notably witnessing the problematization of highly valued features of their professional practice such as autonomy and self-regulation [3,5].

In order that current trends towards collaborative practices are successful in the healthcare field, across disciplinary as well as organizational boundaries, there is the need to construct legitimated health professional role identities: “Legitimizing a new identity is a form of institutional work important to institutional change. The construction of professional role identities is particularly critical because identities describe the relationship between an actor and the field the actor operates within” [5, p. 56]. Due to their traditional privileged position in the power-knowledge healthcare hierarchy, this situation is particularly challenging for physicians.

Despite its importance, there is however a dearth of studies exploring the interplay between collaboration and professional role identity in the healthcare sector [6,7,8]. More specifically, very little is known about identity issues in collaborative practices between family physicians (or medical general practitioners) and specialists across organizational boundaries.

Our aim in this paper was to provide meaningful answers to two important calls in the literature. The first is to strengthen our understanding of medical collaboration among levels of care. As noticed by Martin et al., “rare are studies examining the reconstruction of professional boundaries by actors on the ground in the face of technological, managerial or policy changes. Another, under-researched area is the intra-, rather than inter-, professional boundaries within occupations” [6, p. 3]. The second one asks for paying attention to the individual’s subjective experience of different forms of professional work [7]. In this regard, Chreim et al., state, “the professional aspect of roles and identities has received little attention. Further, studies that focus on professional models generally demonstrate a macrosociological perspective and tend to ignore the individual dynamics associated with professional role identity” [8, p. 1517]. More specifically, this study was guided by the following research questions: How do family physicians and specialists enact their professional identity when interacting in their clinical situated context?

## Theory and methods

### Collaboration: some insights

Wood and Gray conceive ‘collaboration’ as what happens “when a group of autonomous stakeholders of a problem domain engage in an interactive process, using shared rules, norms and structures to act or decide on issues related to that domain” [9, p. 146]. Characterized by its autonomy, the medical community seems to be mutating from a craft guild form towards a new collaborative form, the collaborative community, whose distinctive social structures support horizontal coordination of interdependent work processes [7]. Collaborative community is also distinctive in its reliance on value rationality because its members coordinate their activities through a shared commitment to a set of ultimate goals. In the same vein, professionals, including physicians, are called to take organizing more seriously and develop organizational capacities so that professional services become more connected [10].

That said this “mutation” is not accomplished without difficulties. First, the current scant literature on medical collaboration between levels of care delivery clearly points out the significance of power struggles in the reconstruction of professional boundaries. For instance, Currie et al. [11] examined changes in professional relationships among British general practitioners, and between them and medical specialists, following the policy makers’ attribution of a new role to the former “General Practitioner(s) with Specialist Interests” (GPSI). The policy goal was to transfer resources and clinical decision-making power from hospitals to primary care. The authors found that professional relationships were disturbed by this “modernization” of the system through the introduction of the new roles. On the one hand, specialists tried to protect their professional jurisdiction claiming to hold the expertise needed to perform the task adequately, and therefore accepting only subordinate roles for GPSI. On the other hand, GPs were alarmed by the introduction of a “specialized” role (i.e. a new hierarchy) among some of their peers based on a more focused set of competencies and knowledge. These results are congruent with insights from works on the sociology of professions that highlight the importance of the protection and reproduction of professional jurisdictions to the maintenance of professional status and legitimacy [12].

Intertwined with power dynamics, medical collaboration is likewise confronted with identity issues. As stated by Meyer and Hammerschmid [13], public management reforms are also identity projects as they challenge traditional role identities. These identity issues are particularly problematic in health care settings where taken for granted norms, values, about how things should be performed make role identities highly resilient [14].

Role identity is the concept that defines the self in motion, and includes “the goals, values, beliefs, norms, and interaction styles that are typically associated with a role” [15, p. 6]. The way that professionals conceive their role identity is central to how they interpret themselves and behave as such in work situations [8,16,17]. In their quantitative study, Dukerich et al. [18] found that co-operative behaviours between generalists and specialists were positively associated with the attractiveness of their identity and construed image of their organization. From an interpretive/constructivist stance, other scholars have been interested in examining professional role identity construction in the context of interprofessional collaboration [8,14]. However, as suggested above, current literature needs to be developed on processes whereby professional identity dynamics intertwine medical collaboration practices across organizational boundaries.

### Theoretical framework

The construct of identity, applied at the individual or collective levels of analysis, becomes more and more popular in organizational studies [19]. Following a period where identity was conceptualized as a set of enduring characteristics or essences [20], contemporary works emphasize the constructed and processual nature of identity [16,21,22]. Individual identity, which refers to questions such as “Who am I” and “How should I act”, is constituted through comparison and interactions with other people and groups, meaning that how others relate to us is crucial for how we see ourselves [23]. Pushing reflection further, Bucholtz and Hall [24, p. 607] have proposed an interactionist approach to identity that has the benefit of undoing the false dichotomy between structure and agency.

Relying on this approach of identity, our investigation is inspired by the structuration theory [25]. This meta-theory aims at reconciling two prior competing visions of actors’ dynamics: primacy of structures and primacy of actors’ agency. For Giddens, social structures are simultaneously empowering and constraining, and ensue from previous actions. Structures are sets of rules and resources that individuals use and reconstitute in the course of their actions. The position of actors within a particular set of social structures influence the way they use structures to produce/reproduce practices. This reciprocal relationship between agency and structure, coined the duality of social structure, is central to structuration theory. This means that social structures do not have independent existence out of what social agents do in their day-to-day activity [25, p. 26].

Another central premise in structuration theory is that social actors are knowledgeable agents, meaning they are aware of social rules, and possess and apply knowledge in the production and reproduction of every day encounters [25, p. 22]. This competence is expressed in words by means of discursive consciousness, or in routines by means of practical consciousness. That being said, agents are not necessarily fully aware of all the implications of their actions. There are unacknowledged conditions of their actions as well as unintended consequences of them. Agents are also reflexive as they have the capacity to observe and understand what they are doing while they are doing it.

Importantly, social actors’ agency rests for Giddens on their identity, understood as the sense of “continuity across time and space as reflectively interpreted by the agent” [26]. Identity provides an ontological security to agents, “a confidence or trust that the natural and social worlds are as they appear to be” [25, p. 375]. Agents solidify their identity through routines (practical consciousness). When these routines are disturbed, agents will reflect on their actions in an overt strategic way (discursive consciousness). In sum, based on Giddens’ ideas, we view professional identity as a dynamic structural element of social life recursively related to professionals’ congruent actions through sensemaking processes [22].

### The research process

*Context.* In this paper, we report the results of an empirical investigation conducted in the Quebec healthcare delivery system. As in the rest of the country, most healthcare services in this Canadian province are funded by the government through taxpayer contributions, and provided by organizations that offer primary, secondary and tertiary health services. Whereas this is a publicly-funded healthcare system, physicians affiliated with it are autonomous entrepreneurs, either in private practice or working in public institutions, essentially being paid on a fee-for-services basis. Notably, most family physicians in Quebec practice in several diverse, organizational contexts e.g. private clinics and policlinics, community health centres, family medicine units, and departments of family medicine in community hospitals [27]. It is important to mention that, in the Canadian context, “family medicine is struggling for a clear identity. Two divergent directions emerge: preserving all the profession’s traditional functions or concentrating on areas of expertise, and moving towards creating mini specialists” [28].

As it has happened in other Canadian provinces, the Quebec healthcare delivery system has experienced major reforms in the 2000s. Notably in 2004, health decision-makers aimed to replace a provider-oriented logic by a population-based approach. Their main objectives were to: (a) preserve people’s health and consider its determinants, (b) take responsibility for a population residing in a territory, and (c) involve citizens in healthcare decision-making. The first decision they took was to create 95 Health and Social Service Centres (HSSCs) across the province. The new HSSCs resulted from merging several former independent healthcare organizations operating within the same geographical territory, i.e. community health centres, which offered primary health and social services, long-term care institutions, and general acute-care hospitals. Hence, the decision-making power concerning health services organization was decentralized to HSSCs. Having been recently abolished, the regional healthcare agencies were only mandated to support the implementation of health services networks.

At the same time, politicians aimed to reorganize frontline primary medical care through the creation of new family medicine groups of practice (FMGs). Family physicians were then incentivized to work together in FMGs, a new organizational form that overlaps prior contexts of practice [29], which also included nurse practitioners, and deeper collaboration with the rest of the professionals and organizations of the local healthcare network. Besides multidisciplinary teamwork, improving medical collaboration across levels of care delivery was therefore a critical feature of this reform. Collaboration between family physicians and specialists has also been stressed as an important issue by the College of Family Physicians of Canada and the Royal College of Physicians and Surgeons of Canada since 2006. These two professional bodies have made different recommendations in this regard, which include the adoption of new modes of healthcare delivery that privilege professional and organizational collaboration [30].

*Research strategy.* We adopted the case study as a methodological approach. More specifically, we conducted a multiple longitudinal interpretive case study [31] from 2012 to 2015. Each of the three cases involved in the study corresponds to a continuum of care associated with a chronic condition or specific clientele characterized by varying clinical complexity: diabetes (which is a more or less stable illness), mental illnesses (featured by their uncertainty), and frail elderly (generally presenting multi-morbidity). Fieldwork was carried out in the largest HSSC on the island of Montreal (hereafter HSSC-A). Ethics approval for this study was granted by the Research Ethics Board of this organization.

*Participants, data collection and analysis.* We involved 57 participants in this investigation. Adopting a maximum variation purposeful sampling strategy, we first targeted 18 family doctors working in different primary care facilities, and 13 medical specialists (3 endocrinologists, 3 psychiatrists, 2 internists, 1 cardiologist, 1 oncologist, and 1 nephrologist) located in diverse clinical settings.

Family physicians shared with us their views about the most significant continuums of care in their respective caseloads. Fifteen per cent (15%) of them were at the beginning of their career (less than 10 years); 35% at their mid-career (between 10–20 years); and 50% accumulated more than 20 years of clinical practice. Very frequent in the Quebec Healthcare System, most of them (60%) worked in variety of settings, e.g. private clinic and family medicine teaching unit; community health centre, private clinic, and family medicine teaching unit; hospital and community health centre. The remaining 40% of our final sample of family physicians were based on a single organizational setting, e.g. private medical clinic. All of them provided primary medical care to a broad spectrum of patients.

We also included participants other than physicians with the aim to develop a contextualized analysis of intra-medical collaboration (n = 25): eight other health care professionals (seven nurses and one pharmacist); seven decision-makers or counsellors from the regional agency; and 11 HSSC-A managers.

As methods for generating data, we adopted individual face-to-face semi-structured interviews and documentary analysis. We conducted 64 face-to-face semi-structured interviews with the selected 57 participants; we interviewed some of them twice over the research period considered. Individual interviews lasted 25 to 100 minutes. Inspired by the structurationist theoretical framework used in this study, we questioned physicians about their vision (discursive knowledge) of: (1) their professional role and the role of other physicians acting at a different level of care (structure), (2) medical collaboration (agency), (3) issues related to the management of patients (conditions). They were also invited to describe their more or less routinized collaborative practices (practical consciousness), e.g. decision making and information sharing, etc., and their perceptions about possible factors influencing these practices.

During the interviews with family physicians, we also presented and discussed with tem about a clinical scenario (vignette). This strategy, widely acknowledged in the specialized literature for the analysis of professional practices [32], fostered participant’s reflexivity and helped us gather data about possible collaborative practices in the hypothetical clinical situations depicted in the vignette. Finally, we collected several organizational documents that described the plans and interventions for the management of patients suffering from chronic disorders, and the integration of services.

The third and fourth co-authors conducted the interviews, which were immediately after transcribed verbatim by a research administrative staff. Based on the conceptual framework, the entire research team agreed on an initial coding book as initial step for the use of a deductive-inductive thematic analysis [33]. With the support of NVivo 9 software, MAP and LM performed separately an initial open coding on transcribed verbatim from interviews with physicians, which was iteratively refined with the intervention of the rest of co-authors until the definition and description of a consensual list of final themes. Using our conceptual framework, we pursuit the analysis by then characterizing the practices of medical collaboration for each one of the continuum of services (cases). This analysis helped us to develop a taxonomy of collaborative practices as they occur in a physician’s daily practice (cross-case analysis). Subsequently, drawing on interviews with other health professionals and managers, and the collected set of documents, we elaborated interpretations of these practices referring to actors’ identity dynamics in their organizational and institutional organizational context. Our analysis of identity dynamics consisted, for every form of collaboration, to bring out the salient elements of the physicians’ identity that distinguished them from other physicians.

## Results – Professional identity role in medical collaboration

To better understand the enactment of professional identity in intra-medical collaboration, we have broken down the Results section into three subsections. In the first, we describe how medical collaboration was structured in the three continuums of care considered. In the second one, we expose how other health professionals and managers perceive physicians’ roles and their interactions, as well as the impacts of the new structures on collaborative practices. In the last third section, we provide plausible interpretations about the collaborative processes at stake.

### Constructing medical collaboration: Three different possible pathways

Our findings show that, in this HSSC-A, the construction of medical collaboration between family physicians and specialists across boundaries followed different pathways of instantiation.

#### Case #1 – Continuum of services for patients with diabetes

In the case of diabetes, the construction of collaboration between levels of care resulted from an emerging strategy involving the HSSC-A’s managerial team and the regional agency. As diabetes is more prevalent in this area in relation to other territories on the island of Montreal, the HSSC-A suffered from long waiting lists for access to specialized services, which resulted in local managers expressing a keen interest to improve the effectiveness of their services through a better medical collaboration between care levels. Based on the documents gathered during fieldwork, we were able to identify three consecutive phases in the structuration process of medical collaboration around the management of patients suffering from diabetes.

***Phase 1 – Development of the programme (2006–2007).*** A consortium composed of the regional agency and the HSSCs from the territory sends out a call for proposal towards the HSSCs, aiming at developing projects for the management of chronic illnesses. At the HSSC-A, this call was answered by a project proposal of creating a referral centre (RC) in diabetes. The idea of putting in place a Diabetes RC being just defined in general terms, the proposal included the involvement of a medical internist, who soon became the medical leader of the project.

***Phase 2 – Implementation (2007–2009).*** The HSSC-A internist and managers co-operated closely with some representatives of the regional agency in order to determine operational procedures of the RC. A number of family physicians were also approached to better assess their needs. This process resulted in the proposition of a programme based on interdisciplinarity and the formalization of mechanisms of coordination between the actors intervening with diabetic patients. The RC was the major element of this programme involving: (1) an intervention for changing life habits according to which family physicians referred diabetic patients to an interdisciplinary team for a follow up; (2) an educational programme and a tailored treatment that included the participation of the patient to a 3-day intensive training during which he benefitted from the services of an interdisciplinary team, including a specialist. At first, two internists were associated with the RC, but later on a general practitioner who developed a specialized practice in diabetes was added to the medical team, replacing one of the internists. In both cases, the patient benefited from a 2-year RC follow-up.

***Phase 3 – Consolidation (2009 – onwards).*** Several adaptations were brought afterwards by the members of the Diabetes Coordination Committee, in response to needs expressed by the general practitioners of the territory after the implementation of the programme. This is how some particular sectors were developed, e.g. references towards the RC from the emergency room or admission of patients who did not have a regular family physician, as well as an a la carte format, enabling the opportunity to refer the patient for a medical consultation only. Certain procedures were also adopted in order to enable the systemic transmission of information (progress notes) to referring physicians. Importantly, since 2011, the activities of the RC also evolved in a way that this unit handled not only diabetes but other health problems (e.g. osteoporosis), capitalizing then on resources, structures, and directions gained and developed in the diabetes experience.

#### Case #2 – Continuum of services for patients suffering from mental disorders

In the case of mental illnesses, the local team strived to implement the national policy on mental health. As other HSSCs, the HSSC-A faced a number of challenges in mental healthcare delivery, notably the historical lack of collaboration between family physicians and psychiatrists [34]. Here also, we identified three consecutive phases in the structuration process of medical collaboration in mental health.

***Phase 1 – Development of the intervention plan (2005–2010).*** In 2005, the Quebec government launched a major new mental health policy whose aim was to better coordinate medical services across organisational boundaries. One of the measures for doing so was the creation of a waiting list system centralized at the HSSC level. The policy also formalized the role to be played by different health professionals. Specifically, family physicians were designated as central actors in the treatment of patients suffering from moderate mental disorders, and the stabilization of those with serious mental conditions. In order to support family physicians in these new tasks, consideration was given to the role of the respondent psychiatrist and the creation of a multidisciplinary mental health team at the HSSC.

***Phase 2 – Implementation (2011–2013).*** A number of pressing issues in mental healthcare delivery were addressed during this period, notably: (1) a lack of knowledge of the network and of the resources of the territory by family physicians and specialists; (2) a lack of family physicians’ willingness regarding the management of mental health patients; and (3) a culture of working in silos.

Three action avenues were prioritized. The first consisted of a pilot project for improving communication between family physicians and the HSSC-A’s adult mental health team. The second avenue concerned the implementation of the role of a respondent psychiatrist. In 2012, two psychiatrists volunteered for this position. Since then, they have delivered conference presentations, as well as offered meetings and case discussions to medical teams. They both assumed their on-call shifts to offer telephone consultation services. Subsequently, three other respondent-psychiatrists were also appointed sub-specializing in child psychiatry. The last avenue consisted of the management of mental health patients seen at walk-in-clinics and perceived as being “difficult cases”. With regard to the latter, timetables were granted to patients of a targeted walk-in clinic (the largest of the territory), so that the physicians referred them quickly to the HSSSC-A’s mental multidisciplinary team. This service was particularly useful for orphan clienteles who did not have a regular family physician. Family physicians therefore learned over time to have a better knowledge of the HSSC-A resources in mental health.

***Phase 3 – Consolidation (2012 – onwards).*** Multiple improvements were progressively brought to the projects implemented in the prior phase. In this regard, the content of the training offered by the respondent psychiatrists evolved by taking into consideration the feedback of the family physicians and other professionals of the HSSC-A mental health team. Professional roles and coordination mechanisms were also renewed to adjust to the demands of family physicians. For example, to reinforce the newly established medical collaborative practices, the role of liaison nurse in mental health was introduced in one of the clinics of the HSSC-A. Respondent psychiatrists permanently supported her.

#### Case #3 – Continuum of services for frail elderly

From the beginning, and unlike the two other examined cases, the continuum of services for elders did not experience any formal initiative to improve medical collaboration between levels of care. That said, a few efforts were made to respond to certain family physicians’ requests to improve the access to services for frail elderly clienteles living in nursing homes, e.g. timetables for facilitating the access to several acute care hospital services. Consequently, the “frail-elderly” case acts as a case where the organizations were not actively involved in transformations, leaving professionals on their own.

### Other health professionals’ and managers’ gaze on physician’s roles and collaborative practices

Considering that medical collaboration is an important issue in healthcare delivery, the health professionals other than physicians who participated in this investigation, as well as managers, thought that physicians’ roles could be optimized. They suggested that family physicians be case managers of their patients, so responsible for their follow-up; specialists’ interventions should be required only for specific and complex clinical problems:

“My perception of the role of specialist… He does not take care of an elderly person; he manages a pathology of an elderly person. So, uh, I am Mrs. Tremblay, I fall down, I broke my hip. The specialist does a surgery, installs a prosthesis, and does the follow up. When the surgical part and post-surgery is over, he retires. Uh, in chronic diseases, they withdraw less, but the intensity of follow up is not the same as that of the attending physician … uh, the family doctor. They are there to take care of a problem specific to their specialty.” (Organizational manager).

“The role of the family doctor is at the same time … evaluation, orientation and … important roles. Therefore, he must be able to know what is happening in the network to refer the patient to the right places when he needs services. He must be the pivot provider for the patient. The family doctor remains the case manager of the medical case. So our specialists will do their job, but should refer to … family physicians with respect to everything related to family medicine.” (Staff at Health Regional Board).

“Well, a medical specialist is someone who will be able to take the complex problems that could not be solved by the general practitioner. First, a general practitioner should ask for a consultation, informing the specialist about what has been done. The problem should be fixed by the specialist, who should return the patient to the general practitioner to do the follow-up, which is not always done. So we continue to clog the specialists because they maintain the follow-up even if the general practitioner, should normally be able to do the follow up, when the problem is resolved”. (Nurse).

Acknowledging that efforts were deployed to structure medical collaboration between levels of care, some health professionals other than physicians also thought that more should be done for some family physicians to better assume their new roles:

“Because it was perceived as “I refer my patient, you take care of him, and you return him to me when he is well”, many doctors wash their hands. We find that a lot, a lot, a lot. Not all of them, but a big proportion.” (A health professional at the Diabetes centre).

Managers seemed however more positive about the evolution of medical practices around the management of diabetes, although more ambiguous regarding mental services:

“They know each other better and work a lot more as a team. Uh, before, uh … you know, before, family physicians were in their private office and referred… And the patient waited there, and there was no follow-up necessarily: Did he go or not? And the family physician and the specialist said: “We know, send me cases for nothing, send me anything. The file is not ready. I have no idea. Why do you send me that patient? And I cannot join him.” So there was a communication that was flawed between the two groups. Now, since there is a continuity of services, it is much more fluid, the interactions… everyone agreed on common objectives and goals, and a way of working together in the same way, focusing on the needs of the client.” (Organizational manager).

“This is currently the weakest part of our entire organization. That is, too much based on one-off experiences or more, I would say, more local. But it’s like case by case, we are not able to make a structured assessment of the level of collaboration.” (Organizational manager).

### Understanding the enactment of medical collaboration

Our findings from cross-case analysis helped us elucidate the co-existence of a gradient of medical collaborative practices from *quasi-inexistent* through *restrained* to *extended collaboration*. These practices appeared intertwined with three prevalent physicians’ identity roles, namely *medical expert, care coordinator*, and *team member*.

#### Enacting the medical expert identity in quasi-inexistent collaboration

With more or less intensity, the quasi inexistence of medical collaboration between levels of care was ascertained in the three studied cases. Our analysis revealed that this lack of collaboration was intertwined with a particular identity profile of the physicians: they are used to viewing themselves as *medical experts* in their specific scope of practice. In this regard, family physicians who chose not to collaborate in the management of patients with diabetes considered that the care of unstable and complicated diabetes cases fell within the field of general medicine. Indeed, they viewed themselves as utterly competent and equipped to assume their clinical responsibilities. Consequently, they deemed it unnecessary to defer to colleagues choosing to delegate the stable cases to other health care professionals (e.g. nurses) in their environment of practice: “We do not refer our regular patients. We do not need to. Ever. […] It is my job. I am a physician. The patient who decompensates, the dosage of insulin… The physician… That is what he does.” (Family physician).

Similarly, some family physicians who specialized in geriatrics felt competent to assume all of the medical responsibilities related to elders suffering from comorbidities. As such, they were inclined to not appeal to specialists:

“Because it has been such a long time that I have been doing geriatrics, it is not someone that I would send to another doctor because it is someone that I would be able to handle. So, I would not need a specialist to do a diagnosis of dementia. I would be able to do the evaluation, the follow-up, the prescription and the appropriate medication, etc. However, I am going to need an interdisciplinary team around this lady.” (Family physician)

While these physicians did not defer to specialists, they did regularly interact with their fellow family physicians who also had developed a sub-specialisation (e.g. palliative care) and who perceived themselves as medical experts.

Undoubtedly, the organisational context, in particular the existence of a multidisciplinary team in which these family physicians have offered their services, is congruent with their perception of independence towards specialists. Specifically, it has allowed them to dedicate themselves to the management of complex cases and act as experts of medical care within their team. The possibility for these physicians to develop a niche of expertise in their local context has also encouraged the reinforcement of an expert identity:

“You know, the hospital is largely managed by family physicians. Intensive care, it is family physicians. Coronary care, it is family physicians. All of the hospitalisation, except for the surgeries, it is family physicians. The emergency, it is family physicians. Family physicians do the great majority of the hospital work.” (Family physician)

The mental health case investigated showed however that the lack of collaboration might also be wished by specialists. In effect, our data revealed that some psychiatrists who saw themselves as medical experts of the mental health sector were inclined to not be involved in collaborative practices. According to these physicians, the alleged complexity of certain sub-specialities (e.g. child psychiatry) requires experts and a particular approach of care mindful to these patients’ profile. Moreover, these physicians were rather opposed to the function of a respondent physician knowing that in this role they did not need to see a patient to make a diagnosis. The model preconized by policy-makers did not make any sense for them since it counters psychiatrists’ values for practice (dedication, attention, and availability): “I am saying, I am sorry, that it is too complex. Rather than reading a 400 pages file, personally, I would rather see the patient in flesh and blood because we also have the patient’s account. We also have the non-verbal.” Also, they seemed frustrated by the intrusion of administrators in the sphere of health services organisation: “Let psychiatry be ruled by psychiatrists.”

Interestingly, these physicians had a particular vision of collaboration and teamwork coherent with their role identity as experts:

“Well, the multi-team, personally, what I think, is that, hum… it’s to delegate, to trust, and everyone is capable. […] The meetings… The meeting is, that, that is a real cancer… costs a fortune. The evaluation that was made, that is not better with, hum… I could give you examples.” (Psychiatrist)

It also seems that the rather poor concept these specialized physicians have of the role played by family physicians in the mental healthcare delivery somehow comforts them in their decision to not collaborate: “And hum, the young physicians are too much like spoiled babies. Continuity of care, it is not easy because the patients, they do not heal on the first try. To have a charge of continuity, that is tough.” (Psychiatrist)

According to them, family physicians can however play a role in mental health, but limited to the simplest cases that do not require medical collaboration between levels of care:

“Put yourself in the shoes of a family physician. You will not take cases that are too complicated because you will quickly feel overwhelmed. So, you will take simple cases, and when you take simple cases, you do not need a psychiatrist.” (Psychiatrist)

#### Enacting the care coordinator identity in restrained collaboration

Medical collaboration between levels of care can also be articulated in the traditional basic way, i.e. referencing practices. With the medical follow-up of patients traditionally carried out in a parallel and sequential way, the only significant interactions between physicians working at different levels of care appeared to be the written transmission of pertinent information about a particular patient. This form of collaboration was identified mainly in the diabetes and mental health cases. In these clinical contexts, physicians involved in this form of collaboration generally saw themselves as *care coordinators*: “… The main role of the family physician in diabetes care is to be some kind of conductor who organizes, who oversees the follow-up.” (Family physician)

These family physicians also had a particular vision of what the role of specialists should be: they considered the role of the specialist as mainly being an immediate support to the practice of family medicine, an attitude that appears to be at odds with the need of bidirectional exchanges:

“My role it is to detect diabetes as soon as possible […]. And then, well, I have to be available to see them on appointment, and then intervene. Now, that is essential […] There are medical techniques that are very complicated, that request a training of several years, which I do not have. And I am going to appeal to, at that moment, to a specialist. A good specialist, he has to be available. I think that goes without saying […] the most important element, it is availability.” (Family physician)

In the mental health case, the importance given by family physicians to patients’ clinical examination made them refer their patients to psychiatrists via the centralized waiting list system instead of discussing the case with them:

“Do I have the good diagnosis? Because this drug does not have the effects it should have. Besides discussing with the respondent psychiatrist about drug dosage, there is not much more to say. It is hard without… without examining the patient.” (Family physician)

Finally, the findings suggested that, once again, the organisational and institutional context in which physicians operate might encourage the adoption of the care coordinator identity role. For instance, the mandate to use the centralized waiting list system by family physicians to have access to specialized services can be perceived as an obstacle to a joint follow-up because in this way little value seemed to be placed on relationships built on trust and informal collaborative exchanges.

#### Enacting team membership via extended collaboration

Extended collaboration, observed in the three continuums of cases, qualifies the collaborative practices characterized by the presence of direct interactions between family physicians and specialists. For instance, in the mental health case, extended collaboration involved a more frequent use of new mechanisms of co-ordination implemented in the HSSC-A territory such as discussion on cases and telephone consultation with the respondent psychiatrist, and participation in continuing professional development activities.

That said, we did not observe shared decision-making between physicians about patients’ care in any of the investigated cases. Whereas these documented practices did not exactly match Wood and Gray’s [9] definition of collaboration, we however labelled them as extended collaboration since the interactions enabled physicians to strengthen their capacity of action.

Consistently across cases, it appeared that physicians who collaborate more with their fellows saw themselves as “members of a medical community within an interdisciplinary and inter-organizational team”:

“What I particularly like in my actual practice is the team work in the long term care hospital, with nurses, caregivers who tell us a lot because they are always with the patient… And the discussions with the psychiatrist, with the physiotherapist, with the ergo… That is teamwork; that is interesting.” (Family physician)

But at the same time, more collaborative family physicians also seemed to further insist on the importance of differentiating their role from other professionals’, and presented themselves as practitioners of ‘true medicine’. This capacity to clearly position their practice in medical terms could foreshadow a de-complex vision of family medicine favourable to their perception of more equal relationships with the specialists; they would therefore be more inclined to interact with them:

“If you want to run it smoothly, physicians must do a doctor’s job. They do not do paperwork. Personally, when I fill in a form, I sign, I fill it in. Then I tell the patient: ‘Go and see my secretary. She will help you filling it in’. It is not my place to do clerical work. What is more, I do not have to be a nurse.” (Family physician)

These family physicians also seemed to have particular expectations regarding the role of the specialists. They expected the latter to prove a superior competence on certain questions: “… I think that a good specialist should be someone able to push further what I do. For me, if I cannot find a solution, I expect a specialist to look further to find a solution.” (Family physician). Some family physicians who took care of frail elderly also expected that specialists be more sensitive to the importance of having a comprehensive vision of the patient with complex needs.

It is interesting to report that the specialists who interacted more with family physicians seemed to display the same identity role distinguishing them from their specialized fellows: “We will never treat a mental health issue without collaborating with other healthcare professionals. It is impossible. So, there has to be a collaboration. It is essential.” (Psychiatrist). And, they also seemed to have a more positive idea of their colleagues in family medicine: “It is not necessarily because the family physician does not have a lot of knowledge or expertise; a lot of them are well advanced with the treatment of diabetes.” (Internist)

As expected, contexts surrounding extended collaboration played a significant role in the emergence of this identity role. For example, family physicians and specialists working together in the same facility seemed to be more inclined to collaborate across levels of healthcare delivery. Furthermore, it seems that the perceived complexity of the cases, e.g. patients with co-morbidities, which require the intervention of several healthcare providers over time, influenced the emergence of extended collaborative practices. In this clinical context, expert and collaborator roles appeared to be instantiated in a rather balanced way.

## Discussion and Conclusions

The role of collaborator is currently emphasized in medical discourses. Specifically in the Canadian context, competency-based medical education explicitly prescribes seven different roles that physicians have to fulfil in their medical practice: medical expert, communicator, collaborator, leader, health advocate, scholar, and professional [35]. The medical expert role is definitively predominant appearing clearly illustrated in the way it is represented as a sort of daisy where ‘medical expert’ constitutes the centre of the flower and the other six roles are the petals.

This professional discourse co-exists with policy decision-makers’ and healthcare organizational managers’ through which interprofessional and inter-organisational collaboration is promoted to deliver more effective and efficient health care. Accordingly, and without contesting the importance of the medical expert role, one could understand that for managers and policymakers, health professionals’ role as collaborator is foundational for better clinical practice.

The findings of our work depart from these two prescriptive and rather static views. By identifying three dynamic tandem patterns of collaboration-medical identity roles, the major theoretical contribution of our work is precisely that a much more complex and fluid ontology of the phenomena at stake occurs. The existence of a gradient in modes of collaboration between family physicians and specialists appears recursively intertwined with different medical identity roles [25]. What is more, the entanglement of particular identity roles and specific collaborative practices became meaningful through a complex net of organizational and institutional features, and patients’ nosological profiles. Said differently, physicians can enact different role identities (structure) and display a variety of collaborative practices (agency) depending on their perceptions of not only the complexity of patients’ medical needs, but also the particularities of the clinical and organizational contexts in which they operate.

This argument is based on the identity role-collaboration patterns that our study reveals. In the first pattern, *quasi-inexistent collaboration* was associated with a strong *medical expert identity role*. This pattern corresponds to a configuration of self-confident physicians, patients’ manageable and stable medical conditions, and organizational features that support medically powerful positions in the hierarchy of health professions. In effect, when caring for patients with diabetes and in geriatrics, family physicians with a solid sense of medical expertise were definitively reluctant to collaborate with specialists because they consider this practice unnecessary. In the case of mental healthcare, psychiatrists considered themselves as the right medical professionals for adequately dealing with these type of patients.

In all these continuums of services, physicians behaved with a great deal of professional autonomy when perceiving themselves as the right medical expert for specific clienteles. Viewing themselves as the appropriate medical expert, their strong ‘solo’ medical expert identity role clearly prevented intra-professional collaboration. Whilst modulated by physicians’ individual perceptions of themselves and of organizational conditions, this was a rather surprising finding when one could expect that, feeling more confident in their role as medical experts, then more powerful physicians would be less threatened to interact with their peers.

That said, when caring for geriatric patients and people suffering from diabetes, the family physicians’ medical expert identity role and non-collaboration with specialists was also a pattern associated with interprofessional collaboration *within a multidisciplinary clinical team*. Here, family physicians easily displayed the roles of both collaborator and leader while affirming their power position as medical experts vis-à-vis other health professionals. In contrast, psychiatrists’ non-collaborative attitude was associated with their consideration of family physicians as ill-skilled physicians to treat patients with mental disorders; in other words, their attitude was related to their poor consideration of family physicians as ‘experts’ in mental medical care, i.e. a low professional reputation.

In a second pattern, a *restrained collaboration* between family physicians and specialists, which consisted on the traditional patient referral, appeared clearly associated with a strong family physicians’ sense of themselves as *care coordinators*, and their views of specialists as ‘supporters’ of the practice of family medicine. Here it is important to point out that this role was sometimes reinforced by an organizational context in which prevailing bureaucratic rules favoured just referrals between physicians, banning in fact other types of medical professional relationships.

We also recognized a third combination in which an *extended collaboration* was associated with a view of professionals as *members* of inter-professional and inter-organizational clinical teams. Interestingly, family physicians as well as specialists put emphasis in differentiating their medical expertise from other health professionals’ roles in their interactions. At the same time, viewing specialists as their partners, family physicians also had higher expectations with regard to the specialists’ ability to provide more humanistic patient-centred care. Put differently, in such situations, the medical expert identity role was not in contradiction to the role of collaborator with peers as the meaning of the former went beyond an individual expertise to involve ‘team’ clinical practice. This pattern appeared favoured when family physicians and specialists organized themselves in private clinics and policlinics; that is, the joint instantiation of medical expert and collaborator identity roles appeared congruent when sustained by clan mechanisms and not prescriptive bureaucratic norms of collaboration. This further corroborates the importance for these professionals of medical autonomy as well as the significance of informal, day-to-day encounters in the same organizational context in view of fostering the role of collaborators and professional reputation while highlighting medical expertise.

This study provides two additional important insights. The first is that we did not identify any type of intra-medical collaboration that fully fit with Wood and Gray’s [9] definition of clinical collaboration in any of the continuums of care. In other words, the medical collaborative practices never implied shared medical decision-making with respect to a particular group of patients. Medical collaboration was rather limited to administrative issues. This finding further corroborates the huge sense of professional autonomy that has traditionally characterized the medical profession.

Intimately related to this, another finding is that, although its importance varied from one situation to another, the medical expert was the identity role revealed in any family physician-medical specialist interactions. Whereas Giddens’ view of the sense of self is rather individualistic, the pervasiveness of the medical expert identity role is also consistent with his acknowledgement of the strong pressures from contextual conditions (in this case, the institution of medical profession) that individuals experience when trying to maintain a coherent narrative about themselves over time.

This study has not only important implications for theory, but also offers interesting contributions for practice. For physicians and their professional organizations, it shows that the medical expert role is enacted not only when the physician works in solo or is placed at the top of the health profession’s hierarchy in interdisciplinary teams, but may in fact be instantiated in a more co-operative and *flat* way. For policy and managerial decision-makers, our study highlights that collaboration not only is an ‘ideal’ difficult to reach that requires the materialization of an appropriate constellation of individual, organizational and clinical dimensions, but due to its non-negligible transaction costs, might not be the optimal solution to current healthcare delivery challenges in *any* circumstance.

## References

[1] Askim, J, Fimreite, AL, Mosely, A and Pedersen, ALH. One-Stop Shops for Social Welfare: The Adaptation of an Organizational Form in Three Countries. Public Administration, 2011; 89(4): 1451–1468. DOI: 10.1111/j.1467-9299.2011.01933.x

[2] Shortell, SM and Casalino, LP. Health Care Reforms Require Accountable Care Systems. Journal of the American Medical Association, 2008; 300(1): 95–97. DOI: 10.1001/jama.300.1.95 18594045

[3] Adler, PS and Kown, SW. The Mutation of Professionalism as a Contested Diffusion Process: Clinical Guidelines as Carriers of Institutional Change in Medicine. Journal of Management Studies, 2013; 50(5): 930–962. DOI: 10.1111/joms.12003

[4] Muzio, D, Brock, DM and Suddaby, R. Professions and Institutional Change: Towards and Institutional Sociology of Professions. Journal of Management Studies, 2013; 50(5): 699–721. DOI: 10.1111/joms.12030

[5] Goodrick, E and Reay, T. Florence Nightingale Endures: Legitimating a New Professional Role Identity. Journal of Management Studies, 2010; 47(1): 55–84. DOI: 10.1111/j.1467-6486.2009.00860.x

[6] Martin, GP, Currie, G and Finn, R. Reconfiguring or Reproducing Intra-Professional Boundaries? Specialist Expertise, Generalist Knowledge and the ‘Modernization’ of the Medical Workforce. Social Science & Medicine, 2009; 68(7): 1191–1198. DOI: 10.1016/j.socscimed.2009.01.006 19201073

[7] Adler, PS, Kwon, SW and Kecksher, C. Perspective – Professional Work: The Emergence of Collaborative Community. Organization Science, 2008; 19(2): 359–376. DOI: 10.1287/orsc.1070.0293

[8] Chreim, S, Williams, BE and Hinings, CR. Interlevel Influences on the Reconstruction of Professional Role Identity. Academy of Management Journal, 2007; 50(6): 1515–1539. DOI: 10.5465/amj.2007.28226248

[9] Wood, DJ and Gray, B. Towards a Comprehensive Theory of Collaboration. Journal of Applied Behavioral Science, 1991; 27(2): 139–162. DOI: 10.1177/0021886391272001

[10] Noordegraaf, M. Risky Business: How Professionals and Professional Fields (Must) Deal with Organizational Issues. Organization Studies, 2011; 10(32): 1349–1371. DOI: 10.1177/0170840611416748

[11] Currie, G, Finn, R and Graham, M. Professional Competition and Modernizing the Clinical Workforce in the NHS. Work Employment Society, 2009; 23(2): 267–284. DOI: 10.1177/0950017009102858

[12] Abbott, A. The System of Professions: An Essay on the Division of Expert Labor. Chicago: The University of Chicago Press; 1988 DOI: 10.7208/chicago/9780226189666.001.0001

[13] Meyer, R and Hammerschmid, G. Public Management Reform: An Identity Project. Public Policy and Administration, 2006; 21(1): 99–115. DOI: 10.1177/095207670602100107

[14] Reay, T, Goodrick, E, Boch Waldord, S and Casebeer, A. Getting leopards to change their spots: co-creating a new professional role identity. Academy of Management Journal, 2017; 60(3): 1043–1070. DOI: 10.5465/amj.2014.0802

[15] Ashforth, BE. Role Transitions in Organizational Life: An Identity Based Perspective. Mahwah, NJ: Erlbaum; 2001.

[16] Pratt, MG, Rockmann, KW and Kaufmann, JB. Constructing Professional Identity: The Role of Work and Identity Learning Cycles in the Customization of Identity among Medical Residents. Academy of Management Journal, 2006; 49(2): 235–262. DOI: 10.5465/amj.2006.20786060

[17] Weick, KE. Enactment and the Boundaryless Career: Organizing as We Work In: Arthur, MB and Rousseau, DM (eds.), The Boundaryless Career: A New Employment Principle for a New Organizational Era, 1996; 40–57. Oxford: Oxford University Press.

[18] Dukerich, JM, Golden, BR and Shortell, SM. Beauty is in the Eye of the Beholder: The Impact of Organizational Identification, Identity, and Image on the Cooperative Behaviors of Physicians. Administrative Science Quarterly, 2002; 47(3): 507–533. DOI: 10.2307/3094849

[19] Alvesson, M, Lee Aschraft, K and Thomas, R. Identity matters: Reflections on the construction of identity scholarship in organization studies. Organization, 2008; 15(1): 5–28. DOI: 10.1177/1350508407084426

[20] Albert, S and Whetten, D. Organizational identity. Research in Organizational Behavior, 1985; 17: 263–295.

[21] Gioia, DA, Schultz, M and Corley, KG. Organizational identity, image, and adaptative instability. Academy of Management Review, 2000; 25(1): 63–81. DOI: 10.5465/amr.2000.2791603

[22] Pratt, MG. Rethinking Identity Construction Processes in Organizations: Three Questions to Consider In: Schultz, M (ed.), Constructing Identity in and around Organizations. Oxford: Oxford University Press; 2012 DOI: 10.1093/acprof:oso/9780199640997.003.0002

[23] Alvesson, M, Karreman, D and Sullivan, K. Professional service firms and identity In: Empson, L, Muzio, D, Broschak, JP and Hining, B (eds.), The Oxford Handbook of Professional Service Firms, 2015; 403–424. Oxford University Press.

[24] Bucholtz, M and Hall, K. Identity and interaction: A socio-cultural linguistic approach. Discourse studies, 2005; 74(5): 585–614. DOI: 10.1177/1461445605054407

[25] Giddens, A. The Constitution of Society: Outline of the Theory of Structuration. Berkeley: University of California Press; 1984.

[26] Giddens, A. Modernity and Self-Identity: Self and Society in Late Modern Age. Stanford: Stanford University Press; 1991.

[27] Contandriopoulos, AP. Profils de pratique des médecins généralistes du Québec [Practice profiles of general practioners in Quebec]. Research report. Montreal: Université de Montréal; 2001. [in French].

[28] Beaulieu, M-D, Rioux, M, Rocher, G, Samson, L and Boucher, L. Family practice: Professional identity in transition. A case study of family medicine in Canada. Social Science & Medicine, 2008; 67(7): 1153–1163. DOI: 10.1016/j.socscimed.2008.06.019 18644668

[29] Rodriguez, C and Pozzebon, M. The implementation evaluation of primary care groups of practice: A focus on organizational identity. BMC Family Practice, 2010; 11(15). DOI: 10.1186/1471-2296-11-15 PMC2841653 20175911

[30] College of Family physicians of Canada, The Royal College of physicians and surgeons of Canada. Conjoint Discussion paper. Family physicians and other specialists: Working and learning together; 2006.

[31] Stake, RE. The Art of Case Studies. Thousand Oaks: Sage; 1995.

[32] Peabody, JW, Luck, J, Glassman, P, Dresslhaus, TR and Lee, M. Comparison of Vignettes, Standardized Patients and Chart Abstraction – A Prospective Validation Study of 3 Methods for Measuring Quality. Journal of the American Medical Association, 2000; 283(13): 1715–1721. DOI: 10.1001/jama.283.13.1715 10755498

[33] Braun, V and Clarke, V. Using Thematic Analysis in Psychology. Qualitative Research in Psychology, 2006; 3(2): 77–101. DOI: 10.1191/1478088706qp063oa

[34] Fleury, MJ, Bamvita, JM, Farand, L and Tremblay, J. Variables Associated with General Practitioners Taking on Patients with Common Mental Disorders. Mental Health in Family Medicine, 2008; 5(3): 149–160. 22477863 PMC2777568

[35] Frank, JR, Snell, LS and Sherbino, J. Draft CanMEDS 2015 Physician Competency Framework – Series II Ottawa: The Royal College of Physicians and Surgeons of Canada; 2014.

